# Cases of Injury of the Head

**Published:** 1826-09

**Authors:** 

**Affiliations:** St. George's Hospital


### INJURIES OF THE HEAD.

### *Cases of Injury of the Head.* Treated at St. George's Hospital, by Mr. Jeffreys.

#### I. *Case of Deafness after Concussion.*

Samuel Humphries was brought to the hospital, February 8th, 1824, at eight o'clock in the evening, having just before fallen, in a state of intoxication, from his coach-box. There was a small wound in the scalp over the left temporal bone, and blood was oozing from the left ear. The bone was not denuded. He was stupid and insensible, which seemed to be as much the effect of drunkenness as of the accident. The lips of the wound were brought together with sticking-plaster; he was put to bed, and ordered to take a senna draught in the morning.

Feb. 9th.—Had slept soundly throughout the night, and was still heavy, stupid, and drowsy. Did not complain of pain; pupils of the eyes quite sensible to light; pulse quick and full; tongue white.

Twelve ounces of blood were taken from the arm; and he was ordered the following pills and draught:—Hydrargyri Submur., Pulv. Antimonialis, āā gr. v.; Conf. Rosæ, q. s. M. fiant Pilulæ duæ statim sumendæ; et post horas tres capiat Haustum Sennæ.

The pulse sunk after the bleeding, but in the evening it had again risen, and sixteen ounces more were taken away.

On the following day (Feb. 10th,) he was in all respects better:

he had no headache; his tongue was clean, and his pulse quick. He had been well-purged: but he was dull and heavy when spoken to, and it was ascertained that he had lost the power of hearing in both ears. Several blisters were put on in succession behind the ears; he was frequently purged, and kept on low diet, but without much benefit. The wound in the scalp healed by the first intention, but he did not recover the sense of hearing; and towards the end of the month he left the hospital.

II. *Case of Fracture of the Cranium, with Extravasation of Blood on the Dura Mater, on the side opposite to that on which the blow was received.*

Anthony Baldwin, sixty-three years of age, was admitted April 5th, 1824, with a lacerated, contused wound of the scalp, three inches long, over the right parietal bone; the right ear was also lacerated and torn through: the bone was not denuded. The accident had happened by a fall down stairs, and, when picked up, he was insensible. The people brought him immediately to the hospital; and, on his arrival, he had nearly recovered his senses. The house-surgeon brought the lips of the wound together with sticking-plaster, and prescribed a Senna Mixture, to be taken every four hours till it operated. He passed a good night; but the next day (April 6th,) he complained of pain in his head, had a dry furred tongue, and his pulse was quick and full.

Twelve ounces of blood were taken from his arm; his bowels were kept open with the Senna Mixture, and low diet prescribed.

Under this treatment the headache was relieved; the wound had a favourable appearance; and every thing seemed to be going on well, until the seventh day, (April 12th,) when he fell into a stupid, half-comatose state; had a dry brown tongue, and a quick but not full pulse.

He was again bled to ten ounces; and had some more purging mixture, which operated freely.

He passed a restless, unquiet night; and on the following day, the eighth from the accident, he continued much in the same state of stupor: his pulse quick and intermitting; his tongue very dry; and he voided his urine involuntarily. Ten ounces more of blood were taken away; soon after which he became delirious, and so violent that three men could scarcely restrain him in bed. A large blister was applied over his head. In the evening, he was more tranquil and rational, and answered questions freely and sensibly. He passed the night more quietly than the preceding, and got some sleep; and the next day was in all respects improved.

On the following day, (April 15th,) he complained of a return of pain in the head; the pupils of the eyes were sluggish and inactive, and his pulse quicker than it ought to have been. He was again bled in the arm to ten ounces; immediately after which his pulse sunk very considerably. In the evening, his tongue was not so dry, and he appeared more sensible. But he had a restless uneasy night.

The next day, (April 16th,) he was still sensible when spoken to, and the wounds in the scalp and ear looked very well and promising: but his pulse was very low, weak, and intermitting; and his tongue dry and brown. In the night, he again became delirious, and was kept in bed with difficulty.

On the 17th, he lay in a dosing, stupid state; breathed with difficulty; and had a quick, feeble, intermitting pulse.

On the 18th, he had a severe attack of erysipelas over the face and head; was very low and exhausted; and in the night he died, being the fourteenth day from the accident.

The body was examined on the following day. On the side of the head opposite to the wound in the scalp, there was found an extensive fissure in the parietal bone, which descended through the temporal bone, and terminated at the cella turcica; it also passed across the sagittal suture to the middle of the right parietal bone. Upon the dura mater, on the left side under the fracture, was a deposit of coagulated blood, of the thickness of a dollar, and as much as two inches in diameter. The dura mater exhibited no signs of inflammation; but the tunica arachnoides was full of vessels carrying red blood; and between it and the pia mater there was a copious effusion of coagulable lymph, and a small quantity of serum. The brain was somewhat firmer than usual.—The upper bone of the sternum was discovered to have been fractured at its junction with the second bone; a circumstance which had not been at all suspected during life.

III. *Case of Lacerated Wound of the Scalp, by which a very large portion of bone was laid bare.*

Admitted December 9th, 1824.—Jacob Littleboy, thirteen years of age, was thrown down by a cart, the wheel of which passed over the side of his head, and inflicted a large lacerated wound in his scalp. When brought to the hospital soon afterwards, the wound was found to extend from the top of his head, in a semicircular direction, across the left parietal bone, towards the base of the skull, and to have separated the scalp from the cranium, so as to lay the bone completely bare for a space as large as the palm of the hand. He had no symptoms of injury of the brain, was perfectly sensible, and had had no sickness. A quantity of gravel and dirt, which had lodged in the wound, was carefully picked out; the edges were brought together with sutures and sticking-plaster, and the whole covered with compresses and bandage, wetted with an evaporating lotion. The boy was put to bed, and ordered a dose of house-physic.

The next day, (Dec. 10th,) he complained of soreness in the head, and the scalp and eyelids were somewhat swollen and puffy; but in other respects he was very well. He had been well purged, and had little or no fever.

On the 11th, the swelling of the head was increased, and the eyelids were closed. On examination, the edges of the wound appeared to have united.

12th.—The edges of the wound had separated, and looked foul and undigested, and the swelling of the scalp had not diminished. His tongue was furred; he was hot, and slightly delirious at times.

A poultice of bread and water was applied to the head; and he was directed to take a grain of Calomel and five grains of Jalap every four hours, until the bowels should be well purged.

This medicine brought away a quantity of dark offensive stools, to the great relief of all his symptoms; and on the following day, (the 13th,) the swelling of the scalp was subsiding, the wound had a more favourable aspect, and he was sensible and nearly free from fever.

On the 15th, the wound was clean and granulating kindly; and on the 19th, the separated portion of the scalp had united to the bone. From this time every thing went on as could be wished; the wound healed very soundly; and he was discharged cured on the 19th of January.

IV. *Case of Fracture and Depression of the Frontal Sinus and the Orbitar Process of the Os Frontis.*

Thomas Aldridge, sixteen years of age, was brought to the hospital, March 28th, 1825, at seven o'clock in the morning, having just before been kicked in the face by a horse which he was cleaning. He was stunned for about five minutes; but soon recovered, and was able to walk to the hospital. On his arrival, he was perfectly sensible and collected; but his countenance was blanched, and his pulse scarcely perceptible from loss of blood. On examination, there was a semicircular wound, corresponding to the shape of the horse's shoe, across the right eyebrow and root of the nose, of about one inch and a half in extent. The ossa nasi were broken to pieces and driven inwards; and there was a fracture of the superciliary ridge, extending into the frontal sinus and within the orbit, with very slight depression of the orbital portion of the superciliary ridge. The eye appeared to be rather more prominent than the other, and the pupil was somewhat larger, but it contracted readily on exposure to light. On sponging the wound before it was dressed, a small portion of medullary matter was perceived, about as big as half a pea. He had lost a good deal of blood from the wound and from the nose, but the hemorrhage was now stopped.

The wound was dressed lightly with lint and sticking-plaster, over which were placed compresses wetted with Spirit Lotion. He was put to bed; and ordered to take the Senna Mixture at intervals until it should operate.

At two P.M. he complained of pain across the forehead, and was beginning to be restless, but was quite rational: pulse eighty.

Twelve ounces of blood were taken from his arm.

At seven P.M. the bowels had not acted; and he was ordered a dose of Calomel and Jalap, which procured two copious evacuations; and at nine P.M. he had a clean tongue, cool skin, and quiet pulse. He slept nearly all night, but awoke occasionally in a state of agitation and alarm, which was, however, of short duration.

On the following morning, (March 29th,) he was collected and quiet, but averse to answer questions or to be spoken to. He complained of slight pain in the forehead; his tongue was rather dry, and his pulse beat 108 in a minute. He was again bled in the arm, and the purging medicine was repeated. Towards evening he became restless, and began to talk rather incoherently. At half-past six, he suddenly became violent and unmanageable, with oppressed breathing, and immediately expired.

*Dissection.*—The fracture extended through the frontal sinus, and along the superciliary ridge into the orbit. The orbitar process of the os frontis was broken and splintered, and some of the fragments had been driven through the dura mater, and were lodged, as well as some splinters of the æthmoid bone, in the substance of the anterior lobe of the brain. The ossa nasi and æthmoid bone were shattered to pieces.

---

